# Gut Microbiota-Bile Acid Crosstalk Contributes to Meat Quality and Carcass Traits of Tan and Dorper Sheep

**DOI:** 10.3390/ijms26136224

**Published:** 2025-06-27

**Authors:** Lixian Yang, Ran Cui, Zhen Li, Mingming Xue, Shuheng Chan, Pengxiang Xue, Xiaoyang Yang, Longmiao Zhang, Fenghua Lv, Meiying Fang

**Affiliations:** Department of Animal Genetics and Breeding, National Engineering Laboratory for Animal Breeding, MOA Laboratory of Animal Genetics and Breeding, Beijing Key Laboratory for Animal Genetic Improvement, State Key Laboratory of Animal Biotech Breeding, Frontiers Science Center for Molecular Design Breeding, College of Animal Science and Technology, China Agricultural University, Beijing 100193, China; ylx123@cau.edu.cn (L.Y.);

**Keywords:** Tan sheep, Dorper sheep, bile acids, colon microbiota, meat quality traits, carcass traits

## Abstract

Tan sheep outperform Dorper sheep in meat-quality traits, including muscle fiber characteristics and fatty acid composition, while Dorper sheep excel in carcass weight. However, the molecular mechanisms underlying these breed-specific traits, especially gut microbiota–bile acid (BA) interactions, remain poorly understood. As host–microbiota co-metabolites, BAs are converted by colonic microbiota via bile salt hydrolase (BSH) and dehydroxylases into secondary BAs, which activate BA receptors to regulate host lipid and glucose metabolism. This study analyzed colonic BA profiles in 8-month-old Tan and Dorper sheep, integrating microbiome and longissimus dorsi muscle transcriptome data to investigate the gut–muscle axis in meat-quality and carcass trait regulation. Results showed that Tan sheep had 1.6-fold higher secondary BA deoxycholic acid (DHCA) levels than Dorper sheep (*p* < 0.05), whereas Dorper sheep accumulated conjugated primary BAs glycocholic acid (GCA) and tauro-α-muricholic acid (*p* < 0.05). Tan sheep exhibited downregulated hepatic BA synthesis genes, including cholesterol 7α-hydroxylase (*CYP7A1*) and 27-hydroxylase (*CYP27A1*), alongside upregulated transport genes such as bile salt export pump (*BSEP*), sodium taurocholate cotransporting polypeptide (*NTCP*), and ATP-binding cassette subfamily B member 4 (*ABCB4*), with elevated gut BSH activity (*p* < 0.05). DHCA was strongly correlated with *g_Ruminococcaceae_UCG-014*, *ENSOARG00000001393*, and *ENSOARG00000016726*, muscle fiber density, diameter, and linoleic acid (C18:2n6t) (|r| > 0.5, *p* < 0.05). In contrast, GCA was significantly associated with *g_Lachnoclostridium_10*, *g_Rikenellaceae_RC9_gut_group*, *ENSOARG0000001232*, carcass weight, and net meat weight (|r| > 0.5, *p* < 0.05). In conclusion, breed-specific colonic BA profiles were shaped by host–microbiota interactions, with DHCA potentially promoting meat quality in Tan sheep via regulation of muscle fiber development and fatty acid deposition, and GCA influencing carcass traits in Dorper sheep. This study provides novel insights into the gut microbiota–bile acid axis in modulating ruminant phenotypic traits.

## 1. Introduction

As a distinguished local breed in Northwestern Ningxia, China, Tan sheep are renowned for superior meat-quality traits, such as muscle fiber characteristics and fatty acid composition, compared to Dorper sheep. Dorper sheep, a South African hybrid breed known for early maturity and rapid growth, excel in carcass traits such as live weight and carcass weight [1,2]. However, the molecular mechanisms underpinning these breed-specific trait differences remain poorly characterized.

Bile acids (BAs), as host–microbiota co-metabolites, represent critical mediators of microbe–host interactions [3]. Initially, the hepatic metabolism of cholesterol generates primary BAs via enzymes, such as cholesterol 7α-hydroxylase (*CYP7A1*) and 27-hydroxylase (*CYP27A1*). Upon entering the intestine through enterohepatic circulation, approximately 95% of primary BAs are reabsorbed in the ileum, whereas the remaining 5% are converted to secondary BAs by colonic microbiota through bile salt hydrolase (BSH) and various dehydroxylases [3,4]. BAs exhibit dual physiological roles in animals. Primarily, they form micelles to facilitate the digestion and absorption of dietary lipids and fat-soluble vitamins [5]. Additionally, trace amounts of BAs that enter the systemic circulation act as signaling molecules, activating bile acid receptors like farnesoid X receptor (FXR) and Takeda G protein-coupled receptor 5 (TGR5), thereby regulating lipid and glucose metabolism in target organs such as the liver and skeletal muscle [6,7,8]. Preclinical evidence shows BAs supplementation effectively improves meat quality and carcass traits in livestock. For instance, ursodeoxycholic acid supplementation in Wagyu cattle diets enhances intramuscular fat marbling and carcass traits via BA metabolism regulation [9]. Similarly, exogenous BA addition in lamb diets alters fat deposition patterns, reducing subcutaneous and tail fat deposition while enhancing meat yield [10].

Gut microbiota–BA interactions are crucial for regulating host fatty acid metabolism, skeletal muscle protein synthesis, and energy metabolism. Secondary BAs such as deoxycholic acid, produced by gut microbiota, activate the TGR5 and FXR receptors, thereby enhancing mitochondrial oxidative phosphorylation in skeletal muscle and promoting fatty acid β-oxidation [11,12]. In a high-fat diet mouse model, FXR activation reduces total BA levels, inhibits hepatic de novo lipogenesis, and significantly alters the fecal content of monounsaturated and polyunsaturated fatty acids, such as linoleic acid (C18:2) [13]. Primary BAs conjugated with taurine or glycine form bile salts that disrupt microbial cell membranes, leading to protein misfolding and DNA oxidative damage [14]. Gut microbiota with high BSH activity mitigate bile salt toxicity via deconjugation, fostering a favorable niche for short-/medium-chain fatty acid-producing bacteria [15,16]. Additionally, gut microbiota may regulate secondary BA metabolism to activate the FXR-FGF15/19 pathway, influencing skeletal muscle protein synthesis and alleviating atrophy [17,18]. TGR5 activation in skeletal muscle modulates myofiber differentiation and energy metabolism, thereby improving skeletal muscle quality [19]. Collectively, these findings highlight the pivotal roles of gut microbiota–BA crosstalk in regulating host metabolic homeostasis.

Previous studies have identified distinct differences in colonic microbiota composition and function between Tan and Dorper sheep, with Tan sheep showing enhanced secondary BA metabolism [20]. However, the colonic BA profile differences between breeds and the mechanisms by which microbiota-BA crosstalk influences meat-quality and carcass traits remain unclear. This study aims to: (1) characterize colonic BA composition and concentrations between breeds via targeted metabolomics; (2) investigate genetic (host *CYP7A1*, *CYP27A1*) and microbial (BSH) determinants of BA profiles differences; and (3) integrate colonic microbiome and longissimus dorsi transcriptome data to construct a multi-omics network [2,20], elucidating BAs as central regulators in trait formation. By establishing a microbiota–BA–muscle gene–phenotype regulatory framework in ruminants, this work provides theoretical insights for genetic improvement of high-quality sheep breeds.

## 2. Results

### 2.1. Comparative Analysis of Colonic Bile Acid Metabolic Profiles in Tan and Dorper Sheep

**Figure 1:**
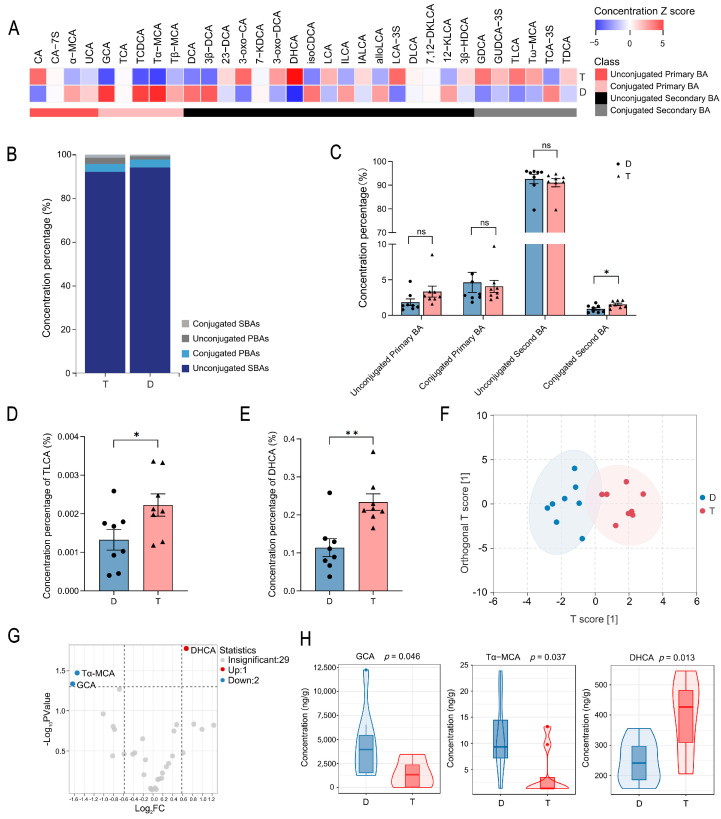
Comparative analysis of colonic bile acid metabolic profiles in Tan and Dorper sheep (*n* = 8 per group). (**A**) Heatmap of Z-transformed relative abundance for bile acid (BA) metabolites in two groups. (**B**) Stacked bar plot depicting colonic BA compositional profiles. (**C**) Statistical comparisons of conjugated vs. unconjugated and primary vs. secondary BA composition ratios in the colon. (**D**,**E**) Quantification of taurolithocholic acid (TLCA) and dehydrocholic acid (DHCA) as proportions of total BAs. (**F**) Orthogonal partial least squares discriminant analysis (OPLS-DA) 2D score plot demonstrating separation of BA profiles between breeds. (**G**) Volcano plot of differentially abundant BA metabolites between groups. (**H**) Violin plot showing the differential BA metabolites between groups. D: Dorper sheep; T: Tan sheep. ns, no significant difference; * *p* < 0.05; ** *p* < 0.01.

Previous studies have demonstrated functional differences in secondary bile acid (BA) metabolism between Tan and Dorper sheep colonic microbiota [20]. To investigate BA metabolism pathways regulated by gut microbiota, we focused on BA metabolites—produced from cholesterol and converted to secondary BAs by gut bacteria [3,4]. Colonic BA profiles were analyzed via ultra-high-performance liquid chromatography-mass spectrometry (UHPLC-MS). The total ion chromatogram (TIC) overlap of quality-control (QC) samples validated data accuracy and reproducibility (Figure S1). Overall, Dorper sheep had 1.5-fold higher total colonic BA concentrations than Tan sheep (Figure S2). A total of 32 BA metabolites were identified and classified into four categories: unconjugated primary BA, conjugated primary BA, unconjugated secondary BA, and conjugated secondary BA (Table S2). A Z-score heatmap visualized the relative variations of BA metabolites between groups (Figure 1A). Proportional analysis showed similar colonic BA compositions across breeds, with unconjugated secondary BAs comprising over 90% of total BAs (Figure 1B). Notably, Tan sheep had a significantly higher proportion of conjugated secondary BAs (Figure 1C). Specifically, taurolithocholic acid (TLCA, a conjugated secondary BA) showed a 1.7-fold higher proportion in Tan sheep than in Dorper sheep (*p* < 0.05, Figure 1D). Dehydrocholic acid (DHCA, an unconjugated secondary BA), was twice as high in Tan sheep (*p* < 0.05, Figure 1E). Orthogonal partial least squares discriminant analysis (OPLS-DA) revealed distinct BA profiles between breeds (Figure 1F), with model parameters (R^2^Y = 0.832, Q2 = 0.413, *p* < 0.05) confirming validity and predictive power (Figure S3). Following filtering by fold change (FC > 1.5 or FC < 0.67) and *p* < 0.05, three BAs showed significant differences between groups (Figure 1G and Table S3). Tan sheep exhibited 1.6-fold higher DHCA (*p* < 0.05), whereas Dorper sheep had 3-fold higher glycocholic acid (GCA, a conjugated primary BA) and 2.9-fold higher tauro α-muricholic acid (Tα−MCA, a conjugated primary BA) (*p* < 0.05), indicating preferential accumulation of conjugated primary BAs in Dorper sheep (Figure 1H).

### 2.2. Influence of Host Genetics and Gut Microbiota on Bile Acid Synthesis, Transport, and Metabolism in Tan and Dorper Sheep

**Figure 2:**
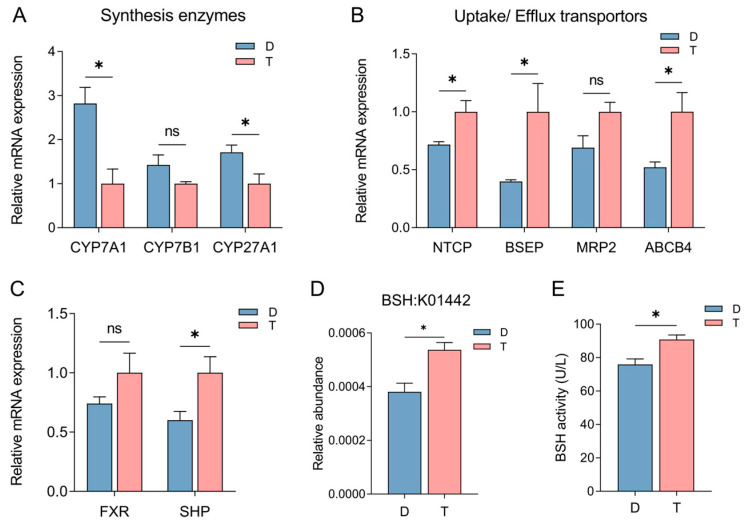
Influence of host genetics and gut microbiota on bile acid synthesis, transport, and metabolism in Tan and Dorper Sheep. (**A**–**C**) The mRNA expression of bile acid synthetic enzymes (*CYP7A1*, *CYP7B1*, *CYP27A1*), transporters (*NTCP*, *BSEP*, *MRP2*, *ABCB4*), and nuclear receptors (*FXR*/*SHP*) in liver tissue (*n* = 4 per group). (**D**) Kyoto encyclopedia of gene and genome orthology (KO) abundance of microbial bile salt hydrolases (BSH) between groups (*n* = 6 per group). (**E**) Colonic BSH activity between groups (*n* = 4 per group). D: Dorper sheep; T: Tan sheep. ns, no significant difference; * *p* < 0.05.

The intestinal BA composition is modulated by hepatic biosynthesis and microbial transformation [21]. To elucidate the molecular mechanisms governing distinct colonic BA profiles between breeds, we quantified hepatic mRNA expression of key BA synthetic enzymes (*CYP7A1*, *CYP7B1*, *CYP27A1*), uptake transporters (*NTCP*/*SLC10A1*) and efflux (*BSEP*/*ABCB11*, *MRP2*/*ABCC2*, and *ABCB4*), and the BA-signaling pathway components (*FXR*/*NR1H4* and *SHP*/*NR0B2*) (Figure 2A–C). Notably, Dorper sheep exhibited significantly higher (*p* < 0.05) expression of *CYP7A1* and *CYP27A1* than Tan sheep (Figure 2A). In contrast, Tan sheep showed upregulated *NTCP*, *BSEP*, and *ABCB4* (*p* < 0.05) (Figure 2B), with *FXR* and *SHP* expression trending upward (Figure 2C). These results indicate that Tan sheep may downregulate *CYP7A1-*/*CYP27A1*-dependent BA biosynthesis via *FXR*/*SHP* signaling.

The intestinal microbiome influences BA profile by converting primary BAs to secondary BAs [3,4]. Building on our prior finding of enhanced secondary BA biosynthesis in Tan sheep [20], we evaluated the abundances of microbial genes involved in secondary BA metabolism via Kyoto Encyclopedia of Genes and Genomes (KEGG) pathways. Notably, the KEGG Orthology (KO) abundance of bile salt hydrolase (BSH, K01442) was significantly higher (*p* < 0.05) in Tan sheep compared to Dorper sheep (Figure 2D). Consistently, using a microbial BSH enzyme-linked immunosorbent assay (ELISA) kit, we detected significantly elevated (*p* < 0.05) BSH activity in Tan sheep colon contents (Figure 2E). These findings provide mechanistic insights into breed-specific BA metabolic regulation via integration of host genetic and microbial pathways.

### 2.3. Spearman Correlation Networks Between Colonic Microbes, Bile Acid Metabolites, Carcass Traits, and Meat Quality in Tan and Dorper Sheep

**Figure 3:**
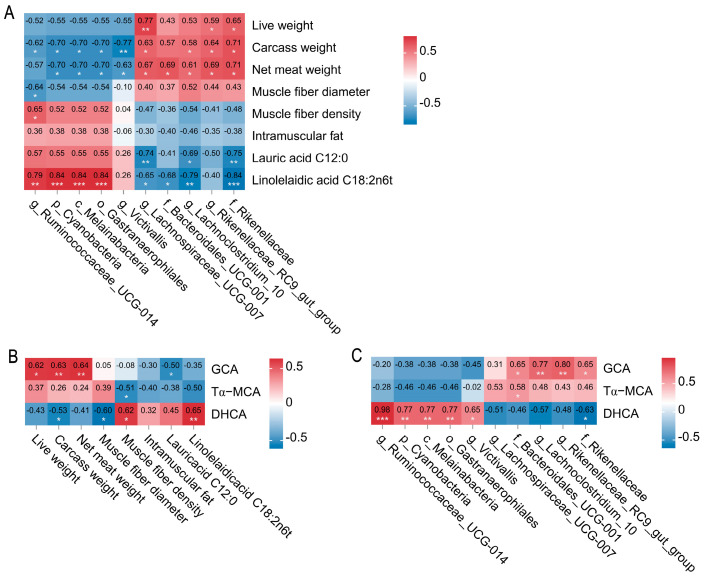
Spearman correlation networks between colonic microbiota, bile acid metabolites, carcass traits, and meat-quality traits in Tan and Dorper sheep. Heatmap of Spearman correlation coefficients and *p*-values: (**A**) between differential microbiota and carcass/meat quality traits; (**B**) between bile acid metabolites and carcass/meat quality traits; (**C**) between differential microbiota and bile acid metabolites. * *p* < 0.05, ** *p* < 0.01, and *** *p* < 0.001.

Phenotypic data on carcass and meat-quality traits of Tan and Dorper sheep, as previously reported [1], highlighted significant inter-breed variations in live weight, carcass weight, net meat weight, muscle fiber diameter, muscle fiber density, intramuscular fat, and specific fatty acids (lauric acid, C12:0; linolelaidic acid, C18:2n6t), detailed in Table S4. Spearman’s rank correlation analysis was performed to assessed associations among colonic microbiota, BA metabolites, and carcass/meat-quality traits. As shown in Figure 3A, the genus *Ruminococcaceae_UCG-014* showed strong positive correlations with muscle fiber density and C18:2n6t, but negative correlations with muscle fiber diameter and carcass weight (|r| > 0.6, *p* < 0.05). Additionally, the phylum Cyanobacteria, class Melainabacteria, and order Gastranaerophilales were significantly positively correlated with C18:2n6t but negatively correlated with carcass weight and net meat weight (|r| > 0.7, *p* < 0.05). Conversely, the genus *Lachnoclostridium_10* and family Rikenellaceae exhibited positive associations with carcass weight and net meat weight but negative associations with C12:0 and C18:2n6t (|r| > 0.5, *p* < 0.05). The genus *Rikenellaceae_RC9_gut_group* was positively correlated with carcass traits (|r| > 0.5, *p* < 0.05). BA metabolites also showed significant correlations with phenotypic traits (|r| > 0.5, *p* < 0.05, Figure 3B). Specifically, DHCA was positively correlated with muscle fiber density and C18:2n6t but negatively correlated with muscle fiber diameter and carcass weight. Moreover, GCA was positively associated with carcass traits but negatively correlated with C12:0.

Gut microbes shape BA profiles through biotransformation, which in turn reshapes microbiota by enhancing the abundance of BA-resistant taxa [22]. We further investigated correlations between differential colonic microbiota and BA metabolites in Tan and Dorper sheep. As shown in Figure 3C, *g_Ruminococcaceae_UCG-014*, p_Cyanobacteria, c_Melainabacteria, and o_Gastranaerophilales were significantly positively correlated with DHCA (|r| > 0.6, *p* < 0.05). In contrast, f_Bacteroidales_UCG-001, *g_Lachnoclostridium_10*, and g_Rikenellaceae_RC9_gut_group showed significant positive correlations with GCA (|r| > 0.6, *p* < 0.05). Notably, f_Rikenellaceae was positively associated with DHCA but negatively correlated with GCA. These findings uncover a microbiota–BA regulatory axis underlying breed-specific variations in carcass and meat-quality traits.

### 2.4. Regulatory Role of Bile Acid Metabolites on Host Skeletal Muscle Transcript Expression

**Figure 4:**
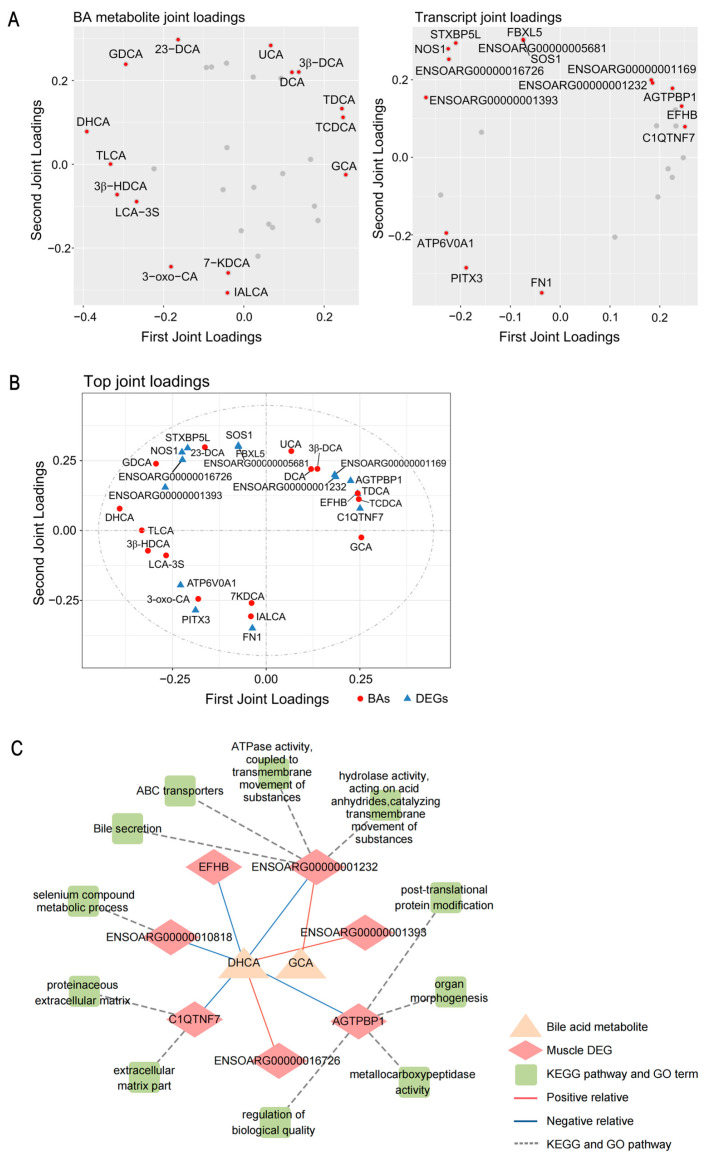
Regulatory role of bile acid metabolites on host skeletal muscle transcript expression. (**A**) Two-way orthogonal PLS (O2PLS) loadings plot for metabolome–transcriptome integration, identified associations between 15 key bile acid (BA) metabolites and longissimus dorsi differentially expressed genes (DEGs), while gray dots denote other unmarked common data points, which provides a comprehensive landscape of multi-omics interactions. (**B**) Bipartite zoom plot of loadings highlighted core interactions, where red dots (BAs) and blue triangles (DEGs) were positioned by covariance strength. Node spacing reflects r, with distant layouts denoting strong negative correlations. (**C**) Network diagram illustrated Spearman correlation between differential BA metabolites, DEGs, and significant enrichment pathway (|r| > 0.8, *p* < 0.05).

Numerous gut microbiota-derived metabolites function as signaling molecules regulating host skeletal muscle metabolism [23,24]. Previous research identified differentially expressed genes (DEGs) in the longissimus dorsi muscle of Tan and Dorper sheep [2]. To systematically investigate associations between BA metabolites and DEGs, we employed orthogonal partial least squares (O2PLS) analysis. Loadings plot (Figure 4A,B) identified the top 15 BA metabolites and DEGs with the strongest correlations in Tan and Dorper sheep. BA metabolome loading revealed several BA metabolites closely related to DEGs, particularly secondary BAs like DHCA and TLCA. Transcriptome loading also identified numerous DEGs associated with BA metabolites, including *ENSOARG00000001393*, *ENSOARG00000016726*, *C1QTNF7*, *EFHB*, and *AGTPBP1*. Spearman’s correlation analysis (Figure S4) and pathway enrichment (Figure S5) further validated the BA metabolite–DEG associations. Network analysis revealed DHCA positively correlated with *ENSOARG00000001393* and *ENSOARG00000016726* genes but negatively correlated with *ENSOARG0000001232*, *C1QTNF7*, *EFHB*, and *AGTPBP1* genes (|r| > 0.8, *p* < 0.05) (Figure 4C). Moreover, GCA showed a strong positive correlation with *ENSOARG0000001232* (r > 0.8, *p* < 0.05). The KEGG and GO annotations indicated these genes were involved in bile secretion, ABC transporters, ATPase activity, hydrolase activity, post-translational protein modification, organ morphogenesis, extracellular matrix part, and others (Figure 4C).

### 2.5. Multi-Omics Integration Analysis Reveals the Effect of Microbiota–Bile Acid–Gene Crosstalk on Carcass and Meat-Quality Traits of Sheep

**Figure 5:**
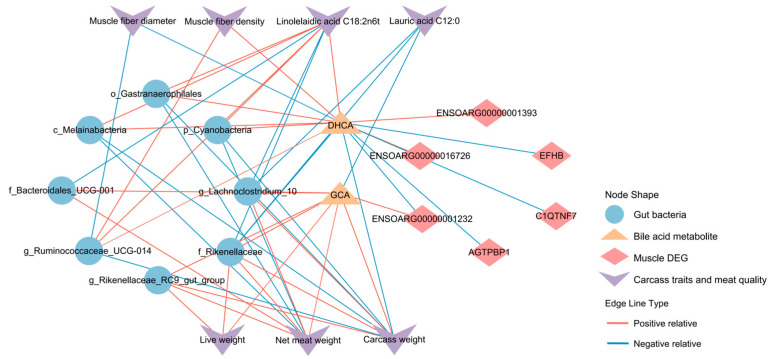
Multi-omics integration analysis reveals the effect of microbiota–bile acid–gene crosstalk on carcass and meat-quality traits of sheep. Spearman correlation depicting interactions among microbial, BA metabolites, differentially expressed genes (DEGs), and phenotypic traits (|r| > 0.5, *p* < 0.05). The shape of nodes: circular, microbial; rectangle, bile acid metabolites; rhombi, DEGs; triangle, phenotypes. The color of the edges: red, positive; blue, negative.

Figure 5 illustrated a correlation network among colonic microbiota, longissimus dorsi DEGs, and carcass/meat-quality traits (|r| > 0.5, *p* < 0.05; Table S5). DHCA exhibited strong positive correlations with p_Cyanobacteria, c_Melainabacteria, and o_Gastranaerophilales, as well as *ENSOARG00000001393* and *ENSOARG00000016726* genes. In contrast, DHCA negatively correlated with *ENSOARG0000001232*, *C1QTNF7*, *EFHB*, *AGTPBP1*, and phenotypically associated with C18:2n6t, carcass weight, and net meat weight. DHCA also positively associated with *g_Ruminococcaceae_UCG-014*, showing phenotypic correlations with muscle fiber diameter, muscle fiber density, C18:2n6t, and carcass weight. Conversely, GCA exhibited positive associations with *g_Lachnoclostridium_10*, f_Rikenellaceae, and *ENSOARG0000001232*, phenotypically correlating with C12:0, carcass weight, and net meat weight. Additionally, GCA associated positively with *g_Rikenellaceae_RC9_gut_group* and *ENSOARG0000001232*, showing positive phenotypic associations with carcass traits.

## 3. Discussion

Previous studies have shown that Tan sheep exhibit superior meat quality, characterized by smaller muscle fiber diameter, higher fiber density, and elevated intramuscular fat (IMF) content [1]. Conversely, Dorper sheep display superior carcass traits, including significantly higher live weight, net meat weight, and carcass weight [1]. These phenotypic differences are modulated by both the host genome and the internal environment, particularly gut microbiota. Gut microbiota metabolize host-derived primary conjugated bile acids (BAs) via bile salt hydrolase (BSH)-mediated deconjugation, releasing free BAs that are converted into secondary BAs [25,26]. Accumulating evidence indicates that these BA metabolites act as signaling molecules, influencing host skeletal muscle quality and function [17,18,19]. However, the underlying mechanisms through which gut microbiota–BA interactions modulate sheep meat and carcass traits remain poorly understood.

Previous studies have identified significant differences in colonic microbial composition between breeds, with Tan sheep showing a significantly enhanced capacity for secondary BA metabolism [20]. BA-targeted metabolomic analysis revealed that total BA content in Dorper sheep was 1.5-fold higher than in Tan sheep. Specifically, Tan sheep exhibited elevated levels of secondary BA deoxycholic acid (DHCA), whereas Dorper sheep accumulated the conjugated primary BAs glycocholic acid (GCA) and tauro-α-muricholic acid (Tα−MCA). KEGG functional annotation and ELISA-based BSH activity assays revealed higher BSH activity in Tan sheep colonic microbiota. As the key enzyme catalyzing primary-to-secondary BA conversion, BSH promotes deconjugation of glycine/taurine-conjugated BAs [16]. Thus, the different colonic BA composition is likely attributed to Tan sheep microbiota with higher BSH activity, which facilitates DHCA precursor supply, whereas Dorper sheep microbiota exhibit lower BSH activity, leading to conjugated primary BA accumulation. Moreover, bile tolerance, crucial for microbiota survival and colonization, is mainly attributed to BSH-mediated bile salt deconjugation [16]. This indicates that Tan sheep microbiota with higher BSH activity may enhance bile tolerance, thereby promoting microbial colonization.

Hepatic BA metabolism shows breed-specific differences that modulate host lipid metabolism. BAs, as pivotal mediators of the gut–liver axis, regulate cholesterol homeostasis, lipid, glucose metabolism, and dietary fat digestion [27,28]. Quantitative analysis revealed that Tan sheep downregulated BA synthesis genes such as cholesterol 7α-hydroxylase (*CYP7A1*) and 27-hydroxylase (*CYP27A1*), while upregulating transport genes such as bile salt export pump (*BSEP*), sodium taurocholate cotransporting polypeptide (*NTCP*), ATP-binding cassette subfamily B member 4 (*ABCB4*), and the *FXR* pathway downstream molecule *SHP*. *BSEP* facilitates bile salt secretion, *NTCP* mediates BA uptake, and *ABCB4* transports phosphatidylcholine [29,30,31]. The *FXR*-*SHP* pathway acts as a critical negative feedback mechanism for BA homeostasis, inhibiting BA-synthesizing enzyme expression and downstream BA synthesis [32,33]. Therefore, Tan sheep likely downregulate the *CYP7A1*/*CYP27A1*-dependent BA biosynthesis indirectly via the *FXR*/*SHP* pathway, reducing total BA content. We propose that Tan sheep reduce hepatic de novo BA synthesis, accelerate enterohepatic circulation, and reduce intestinal BA accumulation to promote IMF deposition. Conversely, Dorper sheep may enhance fat emulsification and skeletal muscle development via intestinal BA accumulation, thereby improving carcass traits.

Emerging evidence highlights that gut microbiota impact muscle quality by modulating BA profiles and indirectly regulating host lipid, glucose, and energy metabolism through BA receptors [17,34]. We constructed a microbiota–BA–gene–phenotype network, revealing that DHCA was closely correlated with muscle fiber characteristics and IMF content, while GCA was associated with carcass traits. In Tan sheep, elevated DHCA levels positively correlated with *g_Ruminococcaceae_UCG-014*, *ENSOARG00000001393*, *ENSOARG00000016726*, muscle fiber density, and C18:2n6t but negatively with muscle fiber diameter. DHCA also positively correlated with p_Cyanobacteria, c_Melainabacteria, o_Gastranaerophilales, and C18:2n6t, but negatively with *ENSOARG0000001232*, *C1QTNF7*, *EFHB*, *AGTPBP1*, carcass weight, and net meat weight. Notably, *g_Ruminococcaceae_UCG-014* and o_Gastranaerophilales belong to the the phylum Firmicutes, whose genera, such as *Clostridium* [35], *Christensenella* [36], and *Lacticaseibacillus* [37], exhibit BSH activity. We propose that *g_Ruminococcaceae_UCG-014* and o_Gastranaerophilales may drive high BSH activity of Tan sheep colonic microbiota, facilitating deconjugation of primary BAs to drive DHCA synthesis. During adipocyte differentiation, expression of the *ENSOARG00000001393* homolog *GIMAP8* increases, suggesting a role in fat accumulation [38]. Additionally, *ENSOARG00000001393* and *ENSOARG00000016726* mediate nucleoside phosphate binding and purine ribonucleotide binding, regulating energy metabolism to modulate muscle fiber diameter and density in Tan sheep [2]. Studies in large-tailed Han sheep show *C1QTNF7* downregulated links to the *PPAR*-signaling pathway, which negatively regulates tail fat deposition [39]. Furthermore, the SNP rs7863248 in *AGTPBP1* correlates with obesity prevalence in humans [40]. Therefore, we propose that key microbiota such as *g_Ruminococcaceae_UCG-014* and o_Gastranaerophilales may convert primary BAs to DHCA through BSH activity. As a signaling molecule, DHCA activates TGR5 receptor in skeletal muscle [19], upregulating *ENSOARG00000001393*/*ENSOARG00000016726* and downregulates *C1QTNF7*/*AGTPBP1*, thereby promoting IMF deposition, resulting in higher C18:2n6t content, smaller muscle fiber diameter, and higher muscle fiber density in Tan sheep. Future research could optimize sheep meat-quality by targeting BSH-active microbiota, such as *g_Ruminococcaceae_UCG-014*.

In Dorper sheep, GCA levels positively correlated with *g_Lachnoclostridium_10*, f_Rikenellaceae, *ENSOARG0000001232*, carcass weight, and net meat weight, but negatively with C12:0. GCA also correlated with *g_Rikenellaceae_RC9_gut_group* and carcass traits, highlighting its role in mediating growth-related phenotypes. Supplementation with probiotic strain *LRH05* or resveratrol enhances *Lachnoclostridium* abundance [41,42]. Fecal microbiota transplantation from treated mice into high-fat diet recipients ameliorates obesity through lipid metabolism regulation [42]. As a member of phylum Bacteroidetes, f_Rikenellaceae shows a strong association with lean body phenotypes (BMI < 25) [43]. In calves, dietary supplementation with antimicrobial peptides and tributyrin increases *Rikenellaceae_RC9_gut_group* abundance, linking to improved growth performance [44] and was consistent with our findings. *ENSOARG0000001232* is highly expressed in Dorper sheep skeletal muscle, mediating bile acid secretion, the ATP-binding cassette transporter pathway, and the cyclic adenosine monophosphate-signaling pathway [2]. Thus, we hypothesize that enriched *g_Lachnoclostridium_10*, f_Rikenellaceae, and *g_Rikenellaceae_RC9_gut_group* in Dorper sheep regulate GCA levels, upregulating *ENSOARG0000001232* to promote muscle growth, reducing IMF accumulation, and enhance carcass traits in Dorper sheep.

Currently, direct evidence of microbial BA metabolism impacting meat-quality and carcass traits remains scarce, and our understanding of how microbially derived secondary BAs affect host traits is limited. Future studies should aim to elucidate the causal links among microbiota, BAs, and host meat quality, thereby providing a stronger theoretical basis for optimizing sheep production.

## 4. Materials and Methods

### 4.1. Animals and Sample Collection

This study utilized Tan and Dorper sheep herd reared at tYu-Bo Meat Sheep Breeding Farm in Ningxia Autonomous Region, China. Each group included eight individuals (four males, four females), consistent with prior studies [20]. All lambs were managed under standardized protocols: constant temperature, humidity, and fixed grazing durations, ad libitum access to a standard corn–soybean starter diet and roughage, including alfalfa and corn stover, and free-choice drinking water. At 8 months of age, lambs were slaughtered in a commercial abattoir using standard procedures after a fasting period of 12 h. The colon was ligated at both ends and removed from the gastrointestinal tract. Tissue collection adhered to standardized protocols: approximately 5 mL of mid-colon contents were aliquoted into cryotubes, snap-frozen in liquid nitrogen, and stored at −80 °C for metabolomic analysis; and liver samples from the right hepatic lobe were processed identically for biochemical assays; and Longissimus dorsi muscle samples (approximately 200 g), harvested from the region between the 3rd and 12th ribs within 30 min post-slaughter, were parallel-processed for comprehensive meat-quality evaluations.

### 4.2. Measurement of Meat-Quality Parameters

The intramuscular fat content was quantified using the Soxhlet extraction method [45]. Fatty acid composition was analyzed according to a previous protocol [46], utilizing an Agilent 7820A gas chromatograph (Santa Clara, CA, USA) with GC-MS detection. For the assessment of muscle fiber diameter and density, longissimus dorsi muscle samples were embedded in an OCT compound and cryosectioned. After H&E staining, 10 random fields were imaged under a Motic BA400 microscope (Motic China Group Company, Guangdong, China), and fiber diameter (μm) and density (N/mm^2^) were measured using Motic Images Plus 3.0 software. Although meat-quality parameters for the two sheep breeds have been previously reported [1], the methodologies were not detailed in the earlier study. Thus, this work supplements these protocols to ensure methodological transparency.

### 4.3. Targeted Metabolomics Analysis of Colonic Bile Acids

Targeted bile acid (BA) metabolomics was employed to assess BA metabolite concentrations in all colon content samples. MetWare Biotechnology Co., Ltd. (Wuhan, China) followed standard processes for sample preparation, extraction analysis, metabolite identification, and quantification [47,48].

#### 4.3.1. Sample Preparation and Extraction

The lyophilized colon contents sample was precisely weighed at 20 mg and transferred to an Eppendorf centrifuge tube. Subsequently, 25 mg of precooled grinding beads and 200 µL of a mixed solvent containing 10 µL of internal standard (V methanol:V acetonitrile = 2:8) were added. The mixture was then placed at −20 °C for 10 min to precipitate proteins. After centrifugation at 4 °C and 12,000 rpm for 10 min, 10 µL of the supernatant were subsequently transferred to an auto-sampler vial for subsequent liquid chromatography-mass spectrometry (LC-MS) analysis.

#### 4.3.2. High-Performance Liquid Chromatography Conditions

The sample extracts were analyzed using an ExionLC AD ultra-high-performance liquid chromatography (UHPLC) system and an electrospray ionization (ESI)-triple quadrupole-linear ion trap (QTRAP) mass spectrometer (MS) (Applied Biosystems, Foster City, CA, USA). The UHPLC system was equipped with a Waters ACQUITY UPLC HSS T3 C18 column (2.1 × 100 mm, 1.8 µm; Milford, MA, USA). The mobile phase consisted of solvent A, pure water with 0.01% acetic acid and 5 mmol/L ammonium acetate, and solvent B, acetonitrile with 0.01% acetic acid. The elution program was as follows: ratio of A:B of 95:5 (*v*/*v*) at 0 min, 60:40 (*v*/*v*) at 0.5 min, 50:50 (*v*/*v*) at 4.5 min, 25:75 (*v*/*v*) at 7.5 min, 5:95 (*v*/*v*) at 10 min, and 95:5 (*v*/*v*) at 12 min [49]. The column temperature was maintained at 40 °C, with a flow rate of 0.35 mL/min, and an injection volume of 1 µL. The effluent was transported to an QTRAP-MS.

#### 4.3.3. ESI-MS/MS

The QTRAP system incorporated both linear ion trap (LIT) and triple-quadrupole (QQQ) scanning. The QTRAP^®^ LC-MS/MS system was equipped with an ESI Turbo Ion-Spray interface, operating in both positive and negative ion mode, and was controlled using Analyst 1.6.3 software. The ESI source operation parameters were as follows: source temperature 550 °C; ion spraying voltage −4500 V (negative pole); curtain gas pressure was set at 35 psi [49]. Quality-control (QC) samples were generated by pooling equal volumes from 16 biological samples, which consisted of colonic content samples from eight Tan sheep and eight Dorper sheep. Three independent QC samples were subsequently prepared, each containing an identical mixture of the pooled matrix. During the LC-MS analysis, one QC sample was inserted between every 10 test samples to comprehensively monitor system stability, ionization efficiency, and data reproducibility.

#### 4.3.4. Qualitative and Quantification

Self-built target standard database MWDB (MetWare Biotechnology Co., Ltd., Wuhan, China) provides the basis for the targeted UHPLC-MS platform, which employs standard substances to identify the structure of metabolites. LC-MS/MS data acquisition and analysis were processed in Analyst 1.6.3 software, and the absolute quantitation of metabolites was achieved using Multiquant 3.0.3 software. Qualitative analysis was performed based on retention time, precursor ion pair information, and secondary spectrum data of detected metabolites. Quantitative analysis was performed by multiple reaction monitoring (MRM) analysis of triple quadrupole mass spectrometry. For each MRM transition, collision energy (CE) and declustering potentials (DP) were optimized [50]. A specific set of MRM transitions was observed by the eluted metabolites for each phase. Each metabolite quantification was conducted by comparing the peak area of the extracted ion chromatograms to standard curves. Results are expressed as ng/g of colon content samples.

#### 4.3.5. Compositional Profiling of Colonic Bile Acids

The BA metabolite profiles were subjected to QC checks and data preprocessing using the MetaboAnalyst 5.0 platform [51]. BA metabolites with over 50% missing values across all samples were eliminated, and the remaining missing values were replaced by one-fifth of the minimum value of each variable. The dataset was then normalized using standard Z-score scaling to ensure comparability across samples.

### 4.4. Colon Bile Salt Hydrolases Activity Analysis

Bacterial bile salt hydrolase (BSH) activity of sheep colon contents was determined by using microorganism BSH enzyme-linked immunosorbent assay (ELISA) Kit (YANJIN, Shanghai, China), following the manufacturer’s protocol. BSH enzyme activity is expressed as U/L. Using previously generated colonic microbiome datasets from Tan and Dorper sheep [20], we performed functional annotation via HUMAnN3 (v3.0.3) against the Kyoto Encyclopedia of Genes and Genomes (KEGG) database, with a focus on quantifying the abundance of BSH (K01442).

### 4.5. RNA Extraction and Real-Time Quantitative PCR

Total RNA from sheep liver tissue was extracted using TRIzol reagent (Invitrogen Life Technology, Carlsbad, CA, USA). From each sample, 1 μg of total RNA was reverse-transcribed into cDNA using FastKing gDNA Dispelling RT SuperMix (TIANGEN, Beijing, China). Real-time PCR was performed using Taq Pro Universal SYBR qPCR Master Mix (Vazyme, Nanjing, China) and Bio-Rad CFX96 Real-Time PCR system (Bio-Rad, Hercules, CA, USA). The PCR cycles consisted of 95 °C for 30 s and 40 cycles of 95 °C for 10 s, 60 °C for 30 s, and 72 °C for 30 s. Target genes were normalized to GAPDH, and the 2^−ΔΔCT^ method was used to calculate gene relative expression [2]. The qPCR primers were shown in Table S1.

### 4.6. Statistical Analysis

Data were presented as means ± standard error of the mean (SEM), and all bar graphs were drawn using GraphPad 9.5. For box plots, the centerline represents the median, while the box limits represent the 25th–75th percentile. For between-breed comparisons of liver gene expression, colonic K02774 (BSH) gene abundances, and colonic BSH activities, normality and homogeneity of variances were tested using Shapiro-Wilk and Levene’s tests, respectively. Parametric data were analyzed by Student’s unpaired *t*-test, while non-parametric or heteroscedastic data were evaluated via the Mann-Whitney U test (*p* < 0.05).

For bile acid metabolite analysis, orthogonal partial least squares discriminant analysis (OPLS-DA) was performed using SIMCA 13.0.2 to identify metabolic patterns differentiating the breeds. Heatmaps of z-scored relative abundances were created with Morpheus software (version 2.3.0) to visualize BA distribution. Significance of BA metabolites was determined by Student’s unpaired *t*-test (for normally distributed data with homogeneous variance) or Mann-Whitney U test in SPSS (version 21.0). Fold change (FC) was calculated as log2-transformed ratio between breeds, with differentially abundant metabolites defined by *p* < 0.05 and FC > 1.5 or < 0.67.

Spearman correlation analysis was conducted to assess pairwise associations among gut microbiome OTUs, BA metabolites, RPKM-normalized muscle differentially expressed genes (DEGs), and meat-quality traits, with significance denoted as * *p* < 0.05, ** *p* < 0.01, and *** *p* < 0.001. For integrative analysis, a two-way orthogonal PLS (O2PLS) model was constructed using the OmicsPLS R package (version 2.1.0) [51], integrating BA metabolite profiles and muscle DEG data. The microbe–BA–DEG–phenotype network was built by incorporating significant correlations (|r| > 0.5, *p* < 0.05). Network visualization was performed using Cytoscape 3.8.2.

## 5. Conclusions

Our study identified distinct breed-specific colonic bile acid (BA) profiles in Tan and Dorper sheep, co-regulated by host genetics and microbial transformation. The secondary BA dihydrocholic acid (DHCA) was critical for Tan sheep’s superior meat quality, while the primary conjugated BA glycocholic acid (GCA) regulated Dorper sheep’s enhanced carcass traits. Compared to Dorper sheep, Tan sheep exhibited downregulated hepatic *CYP7A1*, *CYP27A1,* and upregulated *NCTP*, *BSEP*, and *ABCB4* expression, reducing total BA biosynthesis. Meanwhile, colonic microbiota of Tan sheep displayed elevated bile salt hydrolase (BSH) activity, promoting primary BA deconjugation and DHCA accumulation. *g_Ruminococcaceae_UCG-014* and o_Gastranaerophilales mediated DHCA transformation, regulating host skeletal muscle genes *ENSOARG00000001393* and *ENSOARG00000016726* to impact linolelaidic acid (C18:2n6t) content, muscle fiber density, and muscle fiber diameter. Conversely, *g_Lachnoclostridium_10* and *g_Rikenellaceae_RC9_gut_group* modulated GCA content to regulate host skeletal muscle gene *ENSOARG0000001232*, thereby influencing carcass weight and net meat weight.

## Data Availability

All data supporting the results are included in the publication and additional files. For further requests, please contact the corresponding author.

## Supplementary Materials

The following supporting information can be downloaded at: https://www.mdpi.com/article/10.3390/ijms26136224/s1.

## Abbreviations

The following abbreviations are used in this manuscript:

BA	bile acid
BSH	bile salt hydrolase
DHCA	deoxycholic acid
GCA	glycochenodeoxycholic acid
CYP7A1	cholesterol 7α-hydroxylase
CYP27A1	cholesterol 27-hydroxylase
NCTP	sodium taurocholate cotransporting polypeptide
BSEP	bile salt export pump
ABCB4	ATP binding cassette subfamily B member 4
FXR	farnesoid X receptor
IMF	intramuscular fat
