# Supplementary material for: Gut Microbiota-Bile Acid Crosstalk Contributes to Meat Quality and Carcass Traits of Tan and Dorper Sheep
Source: Int J Mol Sci. 2025 Jun 27;26(13):6224. doi: 10.3390/ijms26136224 (PMC12250145; doi:10.3390/ijms26136224)
Supplement: Supplementary file 1 [file ijms-26-06224-s001.zip › Supplementary Files/Supplementary Materials.docx]

Supplementary Materials for

**Gut Microbiota-Bile Acid Crosstalk Contributes to Superior Muscle Fiber Characteristics and High Lauric Acid Content in Tan Sheep**

Lixian Yang *et al*.

*Corresponding author. Email: meiying@cau.edu.cn

**This PDF file includes:**

Supplementary Text

Figures. S1 to S5

Tables. S1 to S5

**Supplementary Figures**


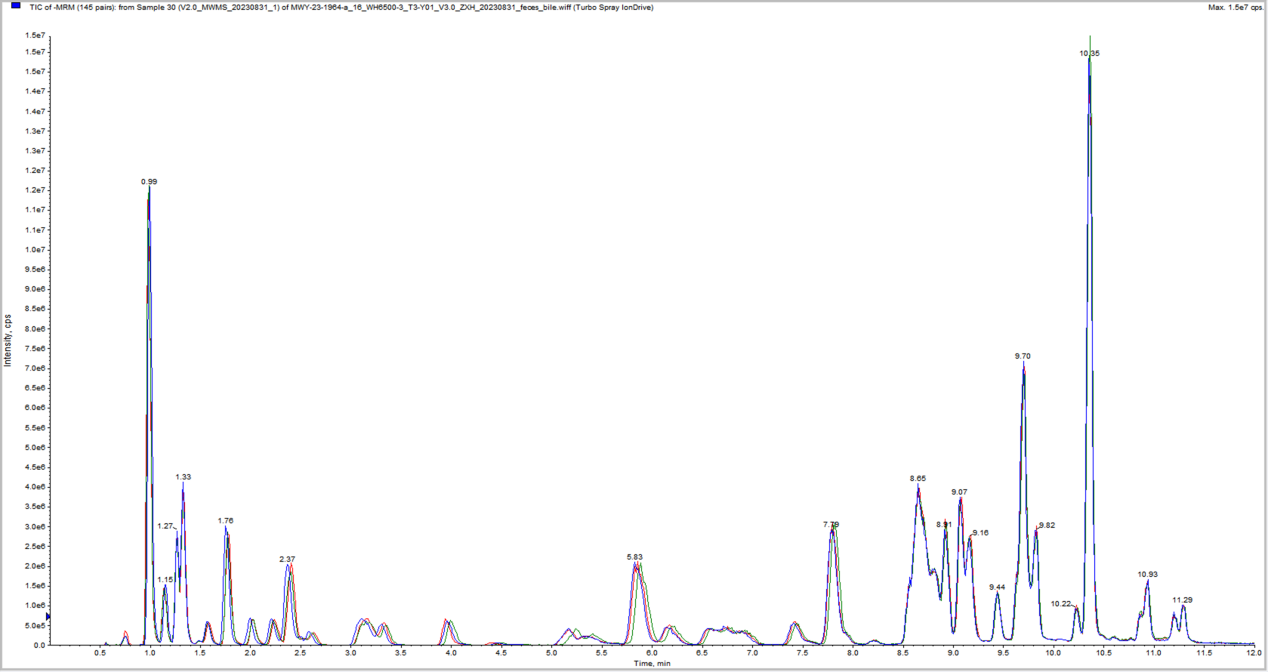


Figure S1. The Quality control-total ion chromatogram (QC-TIC) and sample multi-peak detection diagram.


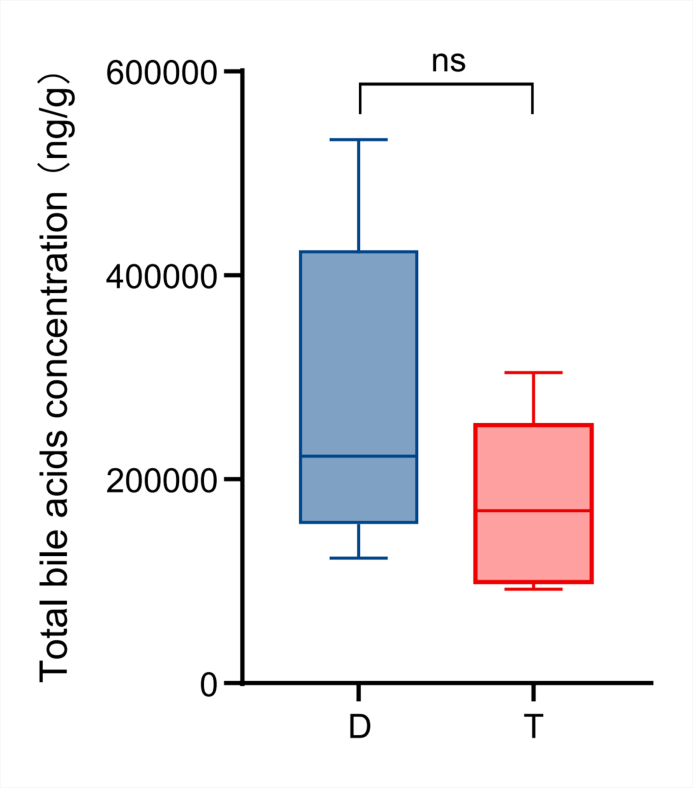


Figure S2. Total concentration of bile acids in colon contents between Tan and Dorper sheep


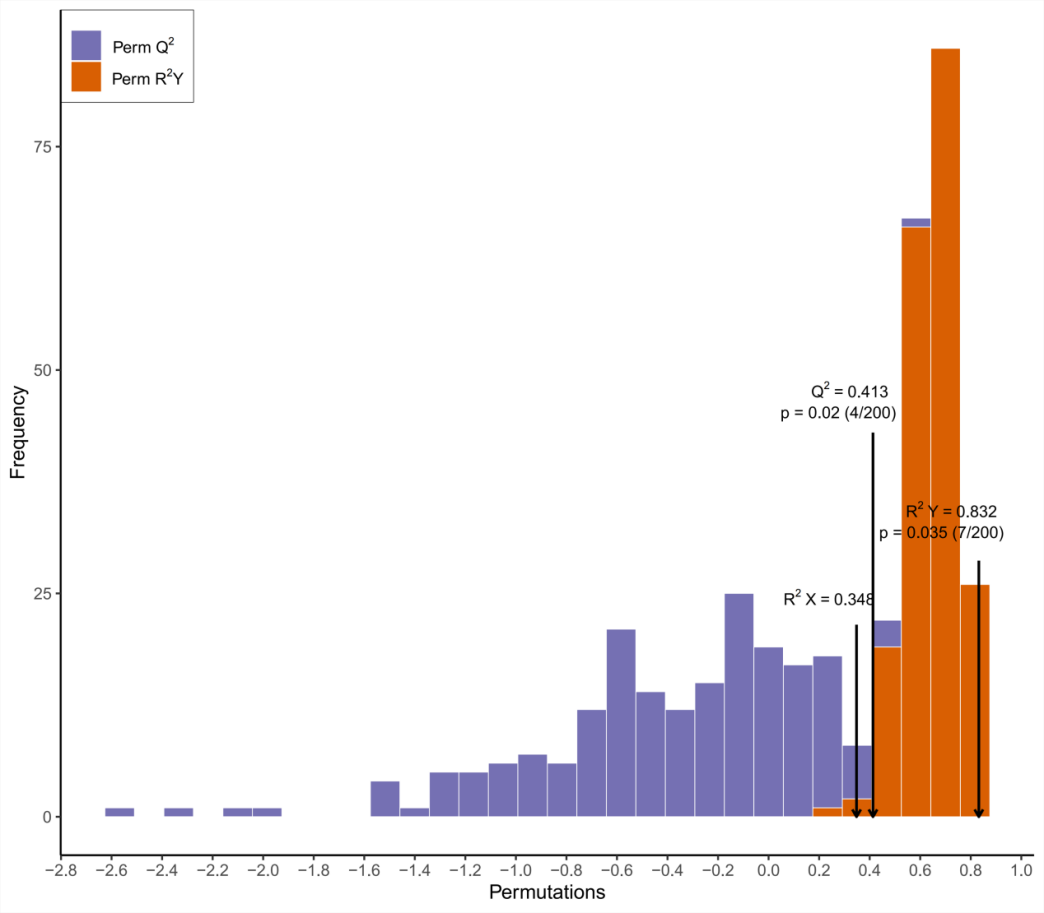


Figure S3. Cross-validation plot of the orthogonal partial least squares discriminant analysis (OPLS-DA) model generated by 200 permutation tests.


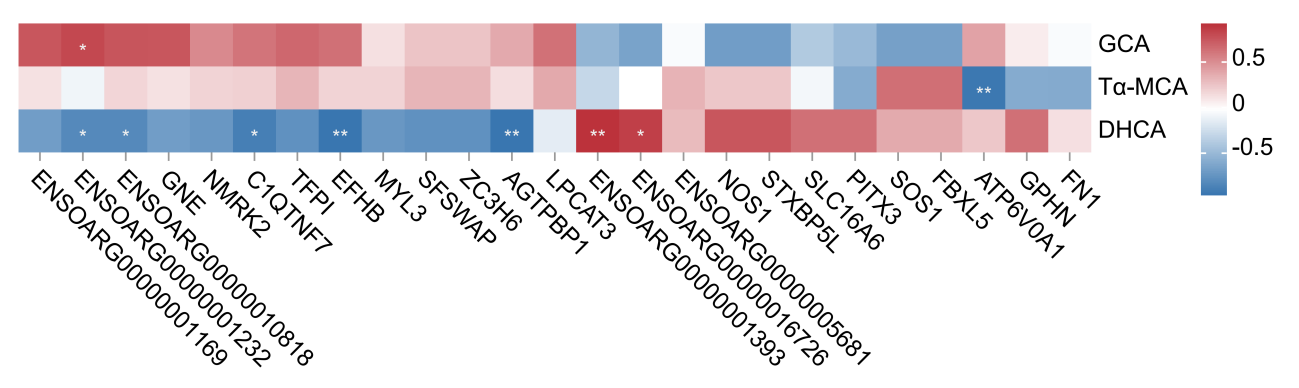


Figure S4. Heatmap of Spearman correlation coefficients between the differential bile acid metabolites and host differentially expressed genes (DEGs), **p* < 0.05, ***p* < 0.01.


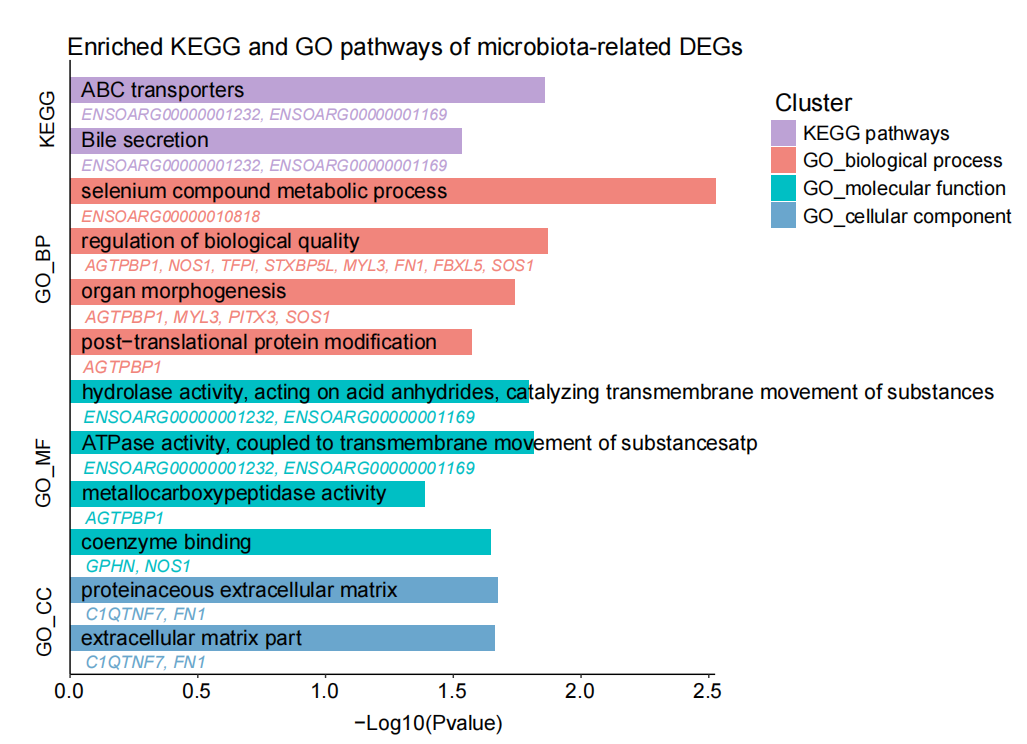


Figure S5. Significantly enriched pathways containing differentially expressed genes (DEGs) associated with gut microbiota of interest. Different colours distinguish KEGG and GO annotations, and gene names are indicated below the pathways.

**Supplementary Tables**

**Table S1: Primer sequences of gene for qRT-PCR.**

(Excel table)

**Table S2. A total of 32 bile acid metabolites were identified and annotated.**

(Excel table)

**Table S3. The results of differential bile acid metabolite screening between Tan and Dorper sheep.**

(Excel table)

**Table S4: The differential phenotypes between Tan and Dorper sheep.**

(Excel table)

**Table S5: Spearman r and p-values for microbiota-longissimus DEGs-meat quality trait correlations in sheep (|r| > 0.5, *p* < 0.05).**

(Excel table)
